# The survival benefit and safety of No. 12a lymphadenectomy for gastric cancer patients with distal or total gastrectomy

**DOI:** 10.18632/oncotarget.7930

**Published:** 2016-03-05

**Authors:** Kun Yang, Hai-Ning Chen, Kai Liu, Wei-Han Zhang, Xin-Zu Chen, Xiao-Long Chen, Zong-Guang Zhou, Jian-Kun Hu

**Affiliations:** ^1^ Department of Gastrointestinal Surgery, West China Hospital, Sichuan University, China; ^2^ Laboratory of Gastric Cancer, State Key Laboratory of Biotherapy, West China Hospital, Sichuan University, China

**Keywords:** gastric cancer, gastrectomy, No.12a lymph nodes, survival, safety

## Abstract

There has still not been a consensus in aspects of survival benefit and safety on No.12a lymph nodes (LNs) dissection for gastric cancer patients. This study was aimed to evaluate this issue for patients with distal or total gastrectomy. Patients were retrospectively divided into 12aD+ group (with No.12a dissection) and 12aD–group (without No.12a dissection). Clinicopathologic characteristics, survival rate, morbidity and mortality were compared. There were 670 patients in 12aD+ group, while 567 in 12aD–group. The baselines between the two groups were comparable. The No.12a LNs metastasis ratio was 11.6% and higher in lower third tumor. The metastasis of No.5 LNs, N stage and M stage were correlated to metastasis of No.12a LNs. There was no difference in morbidity nor mortality between the two groups. The 5-year overall survival rates (5-y OS) were 59.6% and 55.1% in 12aD+ group and 12aD–group respectively (*P* = 0.075). The 5-y OS of patients with negative and positive No.12a LNs were 62.3% and 24.1%. The survival of stage III patients with No.12a positive was better than that of stage IV patients. The 5-y OS were better in 12aD+ group for patients with ages more than 60, lower third tumor, distal gastrectomy, N3 status, or III stages compared with 12aD–group. No.12a lymphadenectomy was independently better prognostic factors for stage III patients. No.12a LNs metastasis should not be considered as distant metastasis. No.12a lymphadenectomy can be performed safely and should be indicated for potentially curable progressive stage tumors requiring distal gastrectomy and might be reserved in patients with stage I or II, or upper third tumor.

## INTRODUCTION

Gastric cancer is the fourth most common cancer and the second leading cause of cancer death worldwide, especially in East Asia [1]. Surgery is the mainstay of treatment for patients with gastric carcinoma and radical lymph nodes (LNs) dissection is an important part of curative resection. Controversy over the lymphadenectomy in the gastric cancer surgery has persisted for several decades. However, the benefiting role of standard D2 LNs dissection for the treatment of gastric cancer has been accepted by a majority of surgeons nowadays [2, 3]. According to the Japanese gastric cancer treatment guideline (3rd English version) [4], D1 or D1+ lymphadenectomy could be performed only for T1N0M0 patients.

No. 12a LNs are defined as the LNs in the hepatoduodenal ligament along the hepatic artery [5]. No.12a LNs metastasis is considered as distant metastasis by 7th American Joint Committee on Cancer (AJCC) classification recently since its significant poor prognosis compared to patients with metastases in the other extraperigastric nodes [6]. However, it is still considered as a regional LNs by the 3rd Japanese classification of gastric carcinoma and 6th AJCC classification, and should be dissected during D2 lymphadenectomy [5, 7]. Actually, the dissection of No.12a LNs which was not included in a D2 lymphadenectomy for lower third gastric cancer according to the general rules for gastric cancer (1st English edition) was reassigned to the extent of D2 lymphadenectomy in the 2nd edition [8]. Even now, the definition of D2 lymphadenectomy in National Cancer Comprehensive Network (NCCN) guideline of gastric cancer (Version 3, 2015) does not include the No.12a LNs dissection [9]. Thus, the group of No.12a LNs has special distinctiveness.

In another hand, the survival benefit of No.12a lymphadenectomy is still controversial and not completely elucidated, although No.12a LNs are required to be dissected in D2 lymphadenectomy when distal or total gastrectomy is performed for advanced or N+ tumors according to the Japanese guideline [4]. Moreover, No. 12a LNs dissection during standard D2 lymphadenectomy is not frequently performed in actual practice. The incidence of No.12a LNs metastasis has been reported from 9–13.1% [10, 11]. The high metastatic incidence supports why No. 12a LNs should be removed in D2 dissection for gastric cancer. Meanwhile, some researches rebutted that No.12a LN metastasis regarded as distant metastasis was inappropriate and its dissection should be included in the extent of D2 lymphadenectomy [12, 13]. Nevertheless, some other researches argued that patients without No.12a lymphadenectomy would not compromise the survival, compared to standard D2 lymphadenectomy [14, 15]. The therapeutic index of No. 12a LNs was only 2.7 for lower third tumor, and zero for upper third tumor [16–18].

Therefore, there still has not been a consensus in aspects of survival and safety on No.12a LNs dissection. The aim of this study is to evaluate the survival benefit and safety of No. 12a lymphadenectomy for gastric cancer patients with distal or total gastrectomy.

## RESULTS

### Patient characteristics

There were 670 patients in the 12aD+ group and 567 in the 12aD–group. The baselines of two groups were comparable, including gender, age, comorbidity, tumor location, resection type, curative degree, differentiation, tumor size, depth of invasion, LNs metastasis, distant metastasis and staging (Table 1). There were 301 patients receiving chemotherapy in the 12aD+ group and 248 in the 12aD–group, without significant different (*P =* 0.676).

**Table 1:** General clinicopathologic characteristics of the patients

	12aD+ group *N =*670 (%)	12aD–group *N =* 567 (%)	*P* value
Gender			0.078
Female	199 (29.7)	195 (34.4)	
Male	471 (70.3)	372 (65.6)	
Age (yrs)			0.288
*<* 60	390 (58.2)	313 (55.2)	
≥ 60	280 (41.8)	254 (44.8)	
Comorbidity	325 (48.5)	300 (52.9)	0.123
Pulmonary	135	120	
Digestive	60	53	
Urological	25	20	
Cardiovascular	110	106	
Endocrinal	70	64	
Neurological	5	3	
Hematological	12	9	
Longitudinal Tumor location			0.169
Upper third	79 (11.8)	46 (8.1)	
Middle third	94 (14.0)	81 (14.3)	
Lower third	481 (71.8)	429 (75.7)	
Whole stomach	16 (2.4)	11 (1.9)	
Circumferential Tumor location			0.109
Lesser curvature	401 (59.9)	346 (61.0)	
Greater curvature	88 (13.1)	61 (10.8)	
Anterior wall	35 (5.2)	47 (8.3)	
Posterior wall	56 (8.4)	51 (9.0)	
Full circle	90 (13.4)	62 (10.9)	
Resection type			0.072
Distal gastrectomy	469 (70.0)	423 (74.6)	
Total gastrectomy	201 (30.0)	144 (25.4)	
Curative degree			0.394
R0	611 (91.2)	509 (89.8)	
R1/R2	59 (8.8)	58 (10.2)	
Differentiation			0.666
G1	14 (2.1)	17 (3.0)	
G2	96 (14.3)	70 (12.3)	
G3	560 (83.6)	480 (84.7)	
Tumor size (cm)			0.099
≤ 2	113 (16.9)	76 (13.4)	
*>* 2, ≤ 5.0	312 (46.6)	252 (44.4)	
*>* 5, ≤ 8.0	185 (27.6)	190 (33.5)	
*>* 8.0	60 (9.0)	49 (8.6)	
Depth of infiltration (T)			0.771
T1	139 (20.7)	106 (18.7)	
T2	73 (10.9)	83 (14.6)	
T3	64 (9.6)	51 (9.0)	
T4a	334 (49.9)	264 (46.6)	
T4b	60 (9.0)	63 (11.1)	
Nodal status (N)			0.770
N0	210 (31.3)	174 (30.7)	
N1	117 (17.5)	106 (18.7)	
N2	100 (14.9)	99 (17.5)	
N3a	154 (23.0)	111 (19.6)	
N3b	89 (13.3)	77 (13.6)	
Distal metastasis (M)			0.490
M0	592 (88.4)	508 (89.6)	
M1	78 (11.6)	59 (10.4)	
Stage			0.574
Ia	109 (16.3)	79 (13.9)	
Ib	49 (7.3)	51 (9.0)	
IIa	40 (6.0)	42 (7.4)	
IIb	84 (12.5)	80 (14.1)	
IIIa	76 (11.3)	64 (11.3)	
IIIb	78 (11.6)	66 (11.6)	
IIIc	156 (23.3)	126 (22.2)	
IV	78 (11.6)	59 (10.4)	

### Metastasis of No.12a LNs

Because the analyses about the metastasis of No.12a LNs, including the metastatic ratio of No.12a LNs, the percentage of patients with positive No.12a LNs, and the correlations between the No.12a metastasis and clinicopathologic factors, didn't involve survival data and surgical data. In order to enroll more patients, we extended the study duration to the June, 2014 for the aforementioned analyses only.

There were totally 84 patients (8.1%) with positive No.12a LNs in 1039 patients who underwent distal or total gastrectomy with No.12a lymphadenectomy. Totally 968 retrieved No.12a LNs with 112 involvement and the metastatic ratio was 11.6% (112/968). The results of metastasis of No.12a LNs according to different tumor locations are summarized in Table 2. The tumor stages were more advanced in patients with positive No.12a LNs, ranking from stage IIIb to stage IV. Seventy four (88.1%) of 84 patients with metastasis in the No.12a LNs had N3 disease.

**Table 2:** No.12a LNs metastasis according to longitudinal tumor location

Metastasis of No.12 LNs	Value
**Metastatic ratio**	
Upper third	6.1% (9/147)
Middle third	7.3% (8/110)
Lower third	13.1% (90/689)
Whole stomach	22.7% (5/22)
Total	11.6% (112/968)
**Percentage of patients with positive No.12a LNs**	
Upper third	4.0% (7/177)
Middle third	6.1% (8/131)
Lower third	9.1% (64/704)
Whole stomach	18.5% (5/27)
Total	8.1% (84/1039)

Several clinicopathologic factors consisting of metastasis status of the No.3, No. 5, No. 7, No. 8a, No. 9 and No. 11p LNs, tumor location, tumor differentiation, tumor size, T stage, N stage and M stage were included in Logistic regression to analyze the correlations to metastasis of No.12a LNs. Results revealed that metastasis of No.5 LNs (*P =* 0.024), N stage (*P =* 0.005) and M stage (*P <* 0.001) were correlated to metastasis of No.12a LNs (Supplementary Table S1).

### Operative variables

The mean number of harvested LNs was significantly higher in 12aD+ group than that of 12aD–group at the cost of prolonged operation time (*P <* 0.001). There were no significant differences in the estimated blood loss (*P =* 0.109) and postoperative hospital stays (*P =* 0.418) between the two groups. There were 2 and 4 patients with reoperations in 12aD+ and 12aD–groups respectively without statistically different (*P =* 0.422). The details can be seen in Table 3.

**Table 3:** Operative variables of the patients

	12aD+ group (*N =* 670)	12aD–group (*N =* 567)	*P* value
No. of harvested lymph nodes(mean ± standard deviation)	30.4 ± 12.3	25.8 ± 12.2	*<* 0.001
Postoperative days (mean ± standard deviation)	11.0 ± 6.2	11.3 ± 7.1	0.418
Estimated blood loss, mL(mean ± standard deviation)	157.6 ± 139.0	172.5 ± 152.3	0.109
Operation time, min(mean ± standard deviation)	248.2 ± 49.8	234.8 ± 56.8	*<* 0.001
No. of patients with reoperation (General anesthesia)	2	4*	0.422

* One patient received double valve replacements because of postoperative infectious endocarditis, rather than postoperative surgical-related complications.

### Morbidity and mortality

The overall postoperative morbidity rates were 14.8% versus 11.8% in the 12aD+ and 12aD–groups without significant different (*P =* 0.128). Neither the constitution of severity of complications nor spectrum of postoperative complications between the two groups was significant different (Table 4). The postoperative mortality was 0.4% versus 0.3% in the 12aD+ and 12aD–groups (*P =* 1.000). Three patients of 12aD+ group and 2 patients of 12aD–group died due to pulmonary failure, anastomotic leakage, and intraluminal hemorrhage.

**Table 4:** Morbidity and mortality

	12aD+ group (*N* = 670)	12aD–group (*N* = 567)	*P* value
Number of patients with morbidity	99 (14.8%)	67 (11.8%)	0.128
Clavien-Dindo classification			
I	31	23	
II	46	21	
IIIa	16	13	
IIIb	2	4	
IVa	0	4	
IVb	1	0	
V	3	2	
Surgical related complications			
Digestive tract hemorrhage	3	0	0.255
Intraperitoneal hemorrhage	1	2	0.596
Digestive tract leakage	2	5	0.257
Wound infection or dehiscence	20	19	0.714
Intraperitoneal infection	8	4	0.382
Intestinal obstruction	3	0	0.255
Gastroparesis	10	8	0.905
Non-surgical related complications			
Pulmonary	35	20	0.149
Renal	0	1	0.458
Digestive	9	3	0.145
Urinary	3	4	0.709
Cardiac	3	4	0.709
Endocrinal	1	1	1.000
Hemostatic	1	1	1.000
Other complications*	3	3	1.000
Mortality#	3 (0.4%)	2 (0.3%)	1.000

* including delirium, skin rash, tinnitus, vertigo, and arthritis.

# due to pulmonary failure, anastomotic leakage, and intraluminal hemorrhage.

### Long-term survival

**Figure 1:**
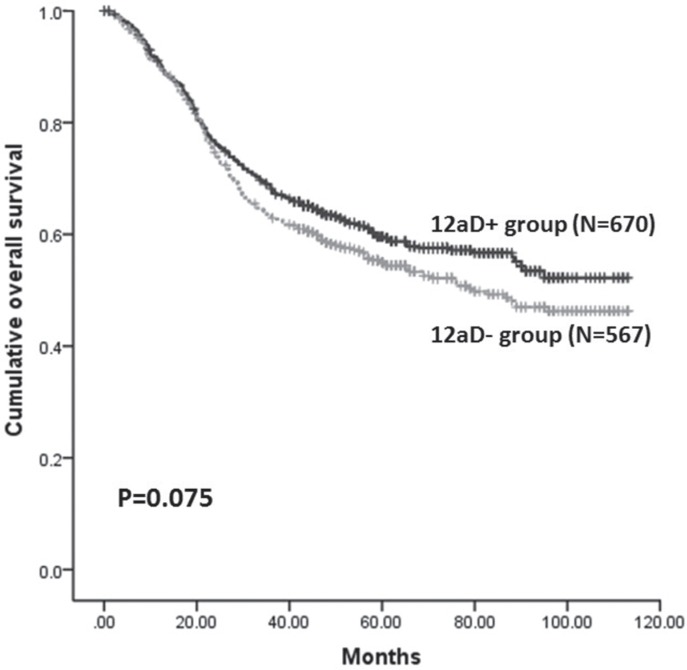
Kaplan-Meier survival analysis of patients between 12aD+ group and 12aD–group for overall patients (P = 0.075)

**Figure 2:**
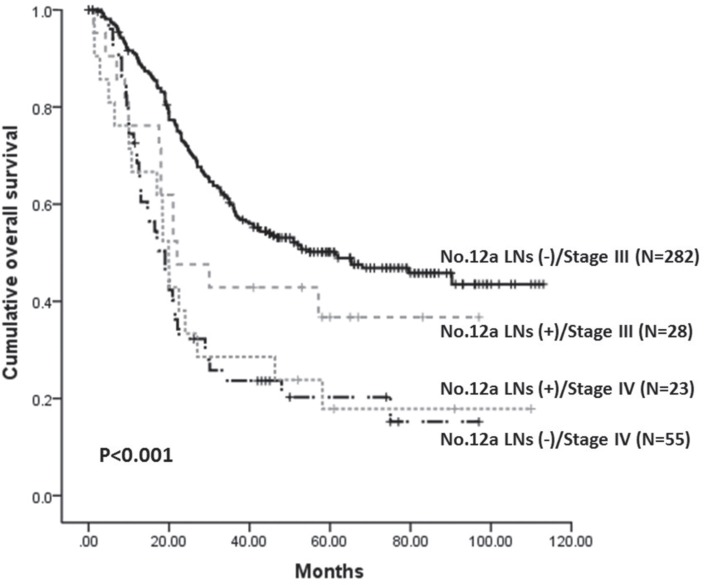
Kaplan-Meier survival analysis of patients with stage III/IV stratified by No.12a LNs metastatic status (P < 0.001)

The 5-year overall survival (5-y OS) for patients with and without No. 12a lymphadenectomy were 59.6% and 55.1%, respectively. Although the 5-y OS was slight better in the 12aD+ group, this was not statistically significant (*P =* 0.075) (Figure 1). Kaplan-Meier analysis showed that 5-y OS of patients with negative and positive No.12a LNs were 62.3% and 24.1% (*P <* 0.001). Because the tumor stages in patients with positive No.12a LNs ranked from stage IIIb to stage IV, we compared the 5-y OS among patients with No.12a LNs negative/stage III, No.12a LNs positive/stage III, No.12a LNs negative/stage IV and No.12a positive LNs/stage IV further. A significantly best 5-y OS was observed in patients with No.12a negative/stage III, compared to other three groups. The 5-y OS did not differ significantly between the patients with No.12a positive/stage IV and patients who had stage IV tumors without No.12a metastasis, both of which were worse than the survival of patients with No.12a positive/stage III (Figure 2).

**Figure 3:**
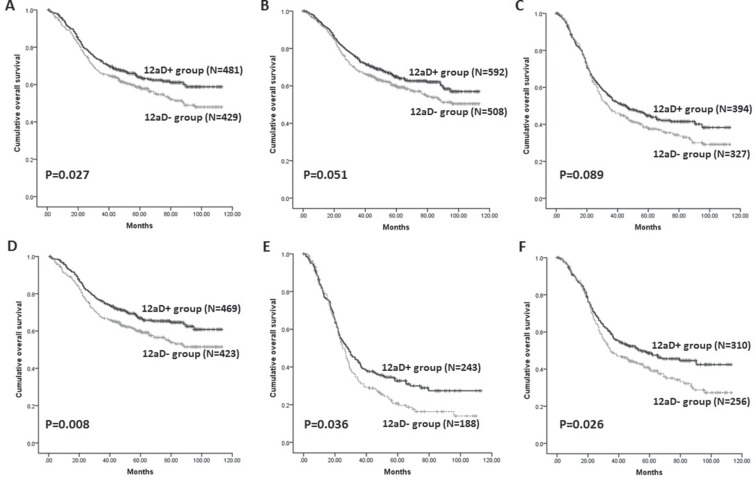
Kaplan-Meier survival analysis of patients between 12aD+ group and 12aD–group stratified by clinicopathologic factors. (A) Patients with lower third tumor (*P =* 0.027); **(B)** Patients with M0 (*P =* 0.051); **(C)** Patients with T4 (*P =* 0.089); **(D)** Patients with distal gastrectomy (*P =* 0.008); **(E)** Patients with N3 (*P =* 0.036); **(F)** Patients with stage III (*P =* 0.026).

When the subgroup analyses were performed stratified by clinicopathologic factors, the 5-y OS were better in 12aD+ group for patients with ages more than 60 (*P =* 0.015), lower third tumor (*P =* 0.027), distal gastrectomy (*P =* 0.008), N3 status (*P =* 0.036), or III stages (*P =* 0.026) compared to those of 12aD–group (Figure 3). The better trend of 12aD+ group also could be found in patients with T4 (*P =* 0.089) and patients with M0 (*P =* 0.051) although no significant differences (Figure 3, Supplementary Table S2). There were no significant differences of 5-y OS for other clinicopathologic factors between the two groups.

### Univariate and multivariate analyses for overall survival

In stage I and II patients, depth of infiltration (*P =* 0.003) and LNs metastasis (*P =* 0.046) were identified as independently associated with survival after adjusting for the clinicopathologic factors (Table 5). And No.12a lymphadenectomy was not an independently survival associated factor. In stage III patients, depth of infiltration (*P =* 0.008), LNs metastasis (*P <* 0.001), curative degree (*P =* 0.004), No.12a lymphadenectomy (*P =* 0.037) and chemotherapy (*P <* 0.001) were identified as independently survival associated factors (Table 5).

**Table 5:** Prognostic factors on the univariate and multivariate analysis

	Stage I and Stage II patients				Stage III patients			
	Univariate HR (95% CI)	*P* value	Multivariate HR (95% CI)	*P* value	Univariate HR (95% CI)	*P* value	Multivariate HR (95% CI)	*P* value
Gender		0.891				0.130		
Male	1				1			
Female	1.03 [0.66–1.61]	0.891			0.82 [0.64–1.06]	0.130		
Age (yrs)		0.086				0.066		
*<* 60	1				1			
≥ 60	1.44 [0.95–2.19]	0.086			1.24 [0.99–1.55]	0. 066		
Longitudinal Tumor location		0.478				0.289		
Upper third	1				1			
Middle third	1.36 [0.36–5.12]	0.653			0.75 [0.50–1.12]	0.158		
Lower third	1.80 [0.57–5.69]	0.320			0.78 [0.57–1.07]	0.120		
Whole stomach	-*	-*			1.16 [0.55–2.45]	0.699		
Circumferential Tumor location		0.083				0.085		
Less curvature	1				1			
Great curvature	0.75 [0.38–1.48]	0.412			1.12 [0.78–1.61]	0.548		
Anterior wall	1.75 [0.94–3.27]	0.080			1.00 [0.60–1.67]	0.997		
Posterior wall	0.65 [0.26–1.63]	0.361			1.49[1.00–2.21]	0.049		
Full circle	1.98 [0.90–4.35]	0.089			1.46 [1.07–2.00]	0.019		
Differentiation		0.396				0.408		
G1	1				-*	-*		
G2	2.20 [0.51–9.49]	0.290			1			
G3	2.53 [0.62–10.34]	0.196			0.86 [0.55–1.34]	0.408		
Tumor size (cm)		0.147				0.074		
≤ 2	1				1			
∼5.0	1.31 [0.79–2.17]	0.298			1.76 [0.82–3.78]	0.145		
∼8.0	2.03 [1.09–3.80]	0.026			2.13 [0.99–4.55]	0.052		
*>* 8.0	0.75 [0.10–5.55]	0.776			2.41 [1.07–5.45]	0.034		
Depth of infiltration (T)		0.005		0.003		0.040		0.008
T1	1		1		-*	-*	-*	-*
T2	2.32 [1.36–3.97]	0.002	1.82 [1.01–3.29]	0.046	1		1	
T3	1.77 [0.85–3.70]	0.126	1.48 [0.69–3.18]	0.312	0.74 [0.36–1.50]	0.398	0.95 [0.46–1.95]	0.881
T4	2.45 [1.40–4.30]	0.002	3.04 [1.68–5.50]	0.000	1.32 [0.76–2.30]	0.330	1.76 [1.00–3.11]	0.049
Nodal status (N)		0.045		0.046		*<* 0.001		*<* 0.001
N0	1		1		1		1	
N1	1.70 [1.05–2.76]	0.032	2.01 [1.14–3.55]	0.017	0.46 [0.14–1.51]	0.202	0.57 [0.17–1.91]	0.360
N2	2.15 [1.09–4.23]	0.027	2.14 [0.97–4.70]	0.059	0.51 [0.16–1.63]	0.257	0.72 [0.22–2.39]	0.592
N3	1.93 [0.47–7.93]	0.362	2.93 [0.69–12.36]	0.144	1.15 [0.37–3.61]	0.805	1.73 [0.53–5.60]	0.361
Curative degree		-*				*<* 0.001		0.004
R0	1				1		1	
R1/R2	-*	-*			2.19 [1.47–3.25]	*<* 0.001	1.84 [1.21–2.80]	0.004
Resection type		0.926				0.152		
Subtotal	1				1			
Total	1.03 [0.55–1.94]	0.926			1.18 [0.94–1.49]	0.152		
No.12a lymphadenectomy		0.527				0.026		0.037
Yes	1				1		1	
No	1.15 [0.75–1.74]	0.527			1.29 [1.03–1.62]	0.026	1.28 [1.02–1.62]	0.037
Chemotherapy		0.342				*<* 0.001		*<* 0.001
No	1				1		1	
Yes	0.81 [0.52–1.25]	0.342			0.63 [0.50–0.79]	*<* 0.001	0.60 [0.47–0.75]	*<* 0.001

* No patients in this subgroup.

## DISCUSSION

The prognostic role of No.12a LNs metastasis and the impact of No. 12a lymphadenectomy on survival and operative safety for gastric cancer patients are still controversial. The previous researches focused on evaluating the difference of OS in patients with or without No.12a LNs metastasis, or compared the D1 or D1+ lymphadenectomy with D2 lymphadenectomy. To our limited knowledge, this is the first study to exclusively investigate the impact of No.12a lymphadenectomy on the survival.

In this research, our results have showed the metastasis ratio of No. 12a LNs was 11.6%, which was in accordance with other researches [10, 11, 19, 20]. And this data indicated that the dissection of No.12a LNs should be paid attention to. Logistic regression showed N stage and M stage were correlated to metastasis of No.12a LNs, which consisted with the finding that patients with positive No.12a LNs ranked from stage IIIb to stage IV and more than 85% of patients with positive No.12a LNs had N3 disease. Lee et al. reported 90% of patients with positive metastasis in the hepatoduodenal ligament LNs had N3 disease [13]. He et al. reported patients with No.12a LNs metastasis had extensive LNs involvement; N stage and M stage were independent predictors of No.12a LNs involvement [12, 21]. In the addition, our results revealed that metastasis of No.5 LNs (*P =* 0.024) was also correlated to metastasis of No.12a LNs, which matched the lymphatic drainage flows of tumor and was supported by other study [12]. It has been reported that metastasis of No.12a LNs was correlated to the tumor location and depth of invasion [10, 22]. Although the frequencies of No.12a LNs metastasis were different according to the tumor locations, tumor location as well as T stage have not been identified as correlated factors in our study and some studies [19, 22]. This discrepancy may partly be caused by relative small number of patients with positive No.12a LNs and number of positive No.12a LNs.

Our results showed the overall 5-y OS was slightly better in the 12aD+ group without statistically different yet, which was in agreement with some researches [14, 15]. But we should notice that there was no No.12a LNs metastasis in stage I and II patients in present study. And it's reasonable that there was no significant different of OS for patients with relative early stage who have extreme low risk of No.12a LNs metastasis between the two groups. Consequently, the survival outcome of whole population would be biased. Therefore, we performed the subgroup analyses stratified by clinicopathologic factors. Our results showed the 5-y OS were better in 12aD+ group for patients with lower third tumor, distal gastrectomy, N3 status, or stage III compared with 12aD–group. In our and other studies, patients with distal third tumors or whole stomach lesion have relative more frequent No.12 LNs metastasis, whereas patients with upper third tumors have least [16, 23, 24]. This may be the reason why 12aD+ group had showed significant better OS for patients with lower third tumor and distal gastrectomy. The fact that there was no significant difference of OS for patients with whole stomach lesion may be caused by the probable type II error. And as our study showed, the patients with positive No.12a LN ranked from stage IIIb to stage IV and most of them had N3 disease. These main partly explain the reason why there was a significant difference of OS for patients with N3 status, or III stages. Moreover, our results also indicated the dissection of No.12a LNs could still bring benefit to the patients even if the No.12a LNs were involved, regardless of stage IV patients. Roukos et al. reported D2 dissection had therapeutic value in patients with No.12a LNs metastases [25]. Multivariate analysis also proved that No.12a lymphadenectomy was independently better prognostic factors for stage III patients, rather than stage I/II patients. Based on the above results, we consider No.12a LNs dissection should be indicated for potentially curable progressive stage tumors requiring distal gastrectomy. Considering the low incidences of No. 12a LNs metastasis, No. 12a lymphadenectomy might be reserved in patients with stage I or II gastric cancer or upper third tumor. The results should be confirmed further in well-designed randomized controlled trials (RCTs). Although comparison of survival between patients with and without No.12a LNs metastasis revealed that those with No.12a LNs metastasis had a significantly poorer survival outcome in our research, patients with distant metastasis had a significant worse overall survival than that of stage III patients with No.12a LN metastasis. Hence, we agree that No.12a LNs metastasis should not be considered as distant metastasis although No.12a LNs metastasis was an important indicator of poor prognosis [12, 13]. However, this result come from a subgroup analysis and should be reviewed with more skepticism.

Although the number of examined LNs can be influenced by several factors, it was associated with the extent of lymphadenectomy [26, 27]. In present study, the mean number of harvested LNs was higher significantly in 12aD+ group than that of 12aD–group. Therefore, there was a concern that dissection of No.12a LNs may lead to a stage migration and could potentially account for survival differences. However, our results have showed that patients with positive No.12a LNs ranked from stage IIIb to stage IV. Most of patients with metastasis in the No.12a LNs had N3 disease. In another hand, the mean number of harvested No.12a LNs in the patients with positive No.12a LNs was 1.4 ± 1.1. In that case, it's very rare for the small mean number to influence the N stage of patients with more than 6 positive LNs. Actually, we have reanalyzed the stage of patients in the 12D+ group provided that we did not count the number of positive No.12 LNs and found that there were only 3 patients downstaged of N stage without change of TNM staging. Even we re-compared the baselines between the two groups using the new stage. There was also no significant difference between the two groups. Therefore, the effect of stage migration caused by the dissection of No.12a LNs could be ignored.

With respect to the safety, our results failed to show that there were significant differences in morbidity and mortality between two groups, which was supported by other study [15]. Kitagawa et al. had reported 2 cases of hepatic infarction resulted from accidental injury of proper hepatic artery in gastric cancer operations [28]. However, No.12a lymphadenectomy related complications, such as injury of proper hepatic artery or portal vein, had not occurred in the two groups. In spite of Galizia et al. showed that patients undergoing total gastrectomy with modified D2 lymphadenectomy (without No.10, 11d and 12a LNs dissection) demonstrated a significant reduction of postoperative morbidity [14]. The routine performance of a splenectomy in this study may account for the increased morbidity in standard D2 group, rather than by the D2 lymphadenectomy itself [29]. Regarding the operation-related variables simultaneously, there were no significant differences in terms of estimated blood loss, length of hospital stay and reoperation rate between the two groups. Hence, we considered No.12a lymphadenectomy can be performed safely with low morbidity and mortality by experienced surgeon with adequate training.

As in any other retrospective studies, limitation of the current analysis includes possible selection bias, detection bias, and performance of analysis bias [30]. However, we have performed subgroup analyses and multivariate analysis to adjust for the shortcomings. In addition, probably there was type II error concerning some subgroup analysis (such as whole stomach subgroup). Anyway, large scale RCTs are needed to explore the survival benefit and safety of No.12a LNs dissection for gastric cancer patients.

In conclusion, No.12a LNs metastasis should not be considered as distant metastasis. No.12a lymphadenectomy can be performed safely and should be indicated for potentially curable progressive stage tumors requiring distal gastrectomy and might be reserved in patients with stage I or II, or upper third tumor.

## METHODS

### Patients

From January 2006 to December 2011, a total of 1237 patients with gastric carcinoma who underwent total or distal gastrectomy were retrospectively analyzed. Patients were divided into 12aD+ group and 12aD–group according to whether No. 12a LNs dissection was performed or not. The preoperative diagnosis of gastric carcinoma was confirmed by gastric endoscopy and biopsy. The exclusion criteria were as follows: ① The patients with other kinds of gastric tumors rather than gastric adenocarcinoma, such as lymphoma, gastrointestinal stromal tumor or adenosquamous carcinoma. ② Patients diagnosed with any previous malignancies. ③ Remnant gastric cancers. The West China Hospital research ethics committee approved retrospective analysis of anonymous data.

### Surgical techniques

In this study, all patients underwent distal or total gastrectomy with D2 or D2 (-No.12a) LNs dissection for gastric cancer [4]. The difference on the extent of lymph node resection was only No.12a LNs between the two groups. The controversial of No.12a lymphadenectomy existed in our institution during the study period. Consequently, although No.12a LNs are required to be dissected in D2 lymphadenectomy according to the Japanese guideline, some doctors did not dissect the No.12a LNs even if for advanced cases when they performed D2 dissection. Billroth I, Billroth II or Roux-en-Y anastomosis with mechanical stapler was performed to reconstruct the digestive tract. For No.12a lymphadenectomy, the anterior layer of hepatoduodenal ligament was opened firstly. After the dissection of No.5 LNs at the root of right gastric artery, the soft tissues which located anterior and interior to the proper hepatic artery were dissected up to the bifurcation of hepatic artery. Then these tissues were retracted leftward and the proper hepatic artery was retracted rightward which could create a surgical plane between the LNs bearing tissues and the proper hepatic artery. Then all the tissues were removed en-bloc along the surgical plane by a combination of blunt and sharp dissection until the exposure of anterolateral wall of the portal vein. Cautious were given to avoid the injury of blood vessels. All the operations were performed by expertise of surgeons specialized in gastrointestinal surgery, at the West China Hospital, Sichuan University.

### Follow-up

Patients underwent a follow up which was done by telephone calls, letters, or outpatient visits. Follow-up assessments were performed every 3–6 months for the first 2 years, every 6–12 months for 3–5 years after surgery and then annually [31]. Fluoropyrimidine alone or fluoropyrimidine/platinum regimens were given to the patients who received postoperative chemotherapy. Overall survival was calculated from the time of surgery until death or the last follow-up for survived patients. As of June, 2015, the overall follow-up rate was 91.4% (1131/1237).

### Clinicopathologic analysis

The clinicopathologic features, such as gender, age, tumor size, tumor location, depth of tumor invasion, LNs metastasis, staging, morbidity, mortality, and survival outcome were collected from the prospective database and compared between 12aD+ group and 12aD–group. The complications were classified according to the Clavien-Dindo Classification [32]. Metastatic ratio of LNs was defined as the ratio of the number of metastatic LNs over the number of harvested LNs. Clinicopathologic terminology was based on the Japanese Classification of Gastric Carcinoma (3rd English version) [5].

### Statistical analysis

SPSS 19.0 software (SPSS, Chicago, IL, USA) was used for statistical analyses. Variables of normality were tested, while confirming the normal distribution, where data are expressed as means ± standard deviation. Two independent *t-*tests for quantitative data and Chi-square test or Fisher's exact test for categorical data were performed, or data was expressed as medians with a range taking the Spearman test into consideration. Survival curves were derived from Kaplan-Meier estimates, and the curves were compared by log-rank tests. The correlation between the No.12a metastasis and clinicopathologic factors was investigated by logistic regression with the forward stepwise (conditional) method. The multivariate regression was performed using the Cox proportion hazards model. Two-sided *p* value less than 0.05 was considered as statistical significance.

## SUPPLEMENTARY MATERIALS TABLES


