# Supplementary material for: The survival benefit and safety of No. 12a lymphadenectomy for gastric cancer patients with distal or total gastrectomy
Source: Oncotarget. 2016 Mar 5;7(14):18750–62. doi: 10.18632/oncotarget.7930 (PMC4951326; doi:10.18632/oncotarget.7930)
Supplement: Supplementary file 1 [file oncotarget-07-18750-s001.pdf]

The survival benefit and safety of No. 12a lymphadenectomy for gastric cancer patients with distal or total gastrectomy

Supplementary Materials

Supplementary Table S1: The correlations between the No.12a metastasis and clinicopathologic factors by logistic regression analysis

	Odds ratio	95% CI	*P* value
Metastasis of No.5 LNs			
No	1		
Yes	3.01	1.15–7.86	0.024
Metastasis of LNs (N stage)			
Less than N3	1		
N3	19.23	2.43–152.10	0.005
Distant metastasis (M stage)			
M0	1		
M1	6.14	2.36–16.02	< 0.001

Supplementary Table S2: Survival analysis stratified by clinicopathologic factors

	12aD+ group (*N* = 670)		12aD–group (*N* = 567)		*P* value
	N	5-y OS	*N*	5-y OS	
Gender					
Female	199	60.2	195	57.2	0.581
Male	471	59.3	372	54.0	0.069
Age (yrs)					
< 60	390	58.3	313	60.3	0.935
≥ 60	280	61.3	254	48.6	0.015
Longitudinal Tumor location					
Upper third	79	42.7	46	39.3	0.969
Middle third	94	59.6	81	49.6	0.424
Lower third	481	64.0	429	58.6	0.027
Whole stomach	16	22.5*	11	18.2*	0.621
Circumferential Tumor location					
Lesser curvature	401	63.6	346	57.7	0.222
Greater curvature	88	67.6	61	61.0	0.089
Anterior wall	35	64.7	47	50.7	0.209
Posterior wall	56	62.2	51	51.0	0.159
Full circle	90	38.4	62	27.6	0.163
Resection type					
Distal gastrectomy	469	67.0	423	60.0	0.008
Total gastrectomy	201	43.5	144	39.6	0.986
Curative degree					
R0	611	63.7	509	59.8	0.097
R1/R2	59	17.4	58	16.4	0.796
Differentiation					
G1	14	-§	17	-§	-§
G2	96	69.7	70	68.2	0.530
G3	560	57.1	480	51.9	0.088
Tumor size (cm)					
≤ 2	113	86.0	76	87.6	0.991
~ 5.0	312	63.7	252	62.2	0.420
~ 8.0	185	44.0	190	40.6	0.526
> 8.0	60	37.2	49	26.1	0.766
Depth of infiltration (T)					
T1	139	89.3	106	90.8	0.648
T2	73	69.4	83	71.3	0.982
T3	64	78.0	51	60.4	0.113
T4	394	44.7	327	38.0	0.089

Nodal status (N)					
N0	210	84.6	174	83.3	0.617
N1	117	70.1	106	64.4	0.319
N2	100	62.2	99	61.5	0.432
N3	243	32.7	188	20.6	0.036
Distal metastasis (M)					
M0	592	64.9	508	59.9	0.051
M1	78	18.9	59	13.1	0.854
Stage					
I	158	89.1	130	89.2	0.915
II	124	75.3	122	71.8	0.473
III	310	48.6	256	40.3	0.026
IV	78	18.9	59	13.1	0.854

*3 year overall survival rate.

§The values could not be calculated due to the small sample size in each subgroup.
